# Influence of Exposure History on the Immunology and Development of Resistance to Human Schistosomiasis Mansoni

**DOI:** 10.1371/journal.pntd.0000637

**Published:** 2010-03-23

**Authors:** Carla L. Black, Pauline N. M. Mwinzi, Erick M. O. Muok, Bernard Abudho, Colin M. Fitzsimmons, David W. Dunne, Diana M. S. Karanja, W. Evan Secor, Daniel G. Colley

**Affiliations:** 1 Center for Tropical and Emerging Global Diseases and Department of Microbiology, University of Georgia, Athens, Georgia, United States of America; 2 Centre for Global Health Research, Kenya Medical Research Institute, Kisumu, Kenya; 3 Department of Pathology, University of Cambridge, Cambridge, United Kingdom; 4 Division of Parasitic Diseases, Centers for Disease Control and Prevention, Atlanta, Georgia, United States of America; George Washington University, United States of America

## Abstract

**Background:**

Previous studies suggest that humans can acquire immunity to reinfection with schistosomes, most probably due to immunologic mechanisms acquired after exposure to dying schistosome worms.

**Methodology/Principal Findings:**

We followed longitudinally two cohorts of adult males occupationally exposed to *Schistosoma mansoni* by washing cars (120 men) or harvesting sand (53 men) in Lake Victoria. Men were treated with praziquantel each time *S. mansoni* infection was detected. In car washers, a significant increase in resistance to reinfection, as measured by the number of cars washed between cure and reinfection, was observed after the car washers had experienced, on average, seven cures. In the car washers who developed resistance, the level of schistosome-specific IgE increased between baseline and the time at which development of resistance was first evidenced. In the sand harvesters, a significant increase in resistance, as measured by the number of days worked in the lake between cure and reinfection, was observed after only two cures. History of exposure to *S. mansoni* differed between the two cohorts, with the majority of sand harvesters being lifelong residents of a village endemic for *S. mansoni* and the majority of car washers having little exposure to the lake before they began washing cars. Immune responses at study entry were indicative of more recent infections in car washers and more chronic infections in sand harvesters.

**Conclusions/Significance:**

Resistance to reinfection with *S. mansoni* can be acquired or augmented by adults after multiple rounds of reinfection and cure, but the rate at which resistance is acquired by this means depends on immunologic status and history of exposure to *S. mansoni* infection.

## Author Summary

Schistosomiasis is a parasitic blood fluke infection of 200 million people worldwide. We have shown that humans can acquire immunity to reinfection after repeated exposures and cures with the drug praziquantel. The increase in resistance to reinfection was associated with an increase in schistosome-specific IgE. The ability to develop resistance and the rate at which resistance was acquired varied greatly in two cohorts of men within close geographic proximity and with similar occupational exposures to schistosomes. These differences are likely attributable to differences in history of exposure to *Schistosoma mansoni* infection and immunologic status at baseline, with those acquiring immunity faster having lifelong *S. mansoni* exposure and immunologic evidence of chronic *S. mansoni* infection. As many conflicting results have been reported in the literature regarding immunologic parameters associated with the development of resistance to schistosome infection, exposure history and prior immune status should be considered in the design of future immuno-epidemiologic studies.

## Introduction

*Schistosoma mansoni* age-infection curves in endemic human populations characteristically show a peak prevalence in children and early adolescence and then a decline beginning in the late teenage years to lower levels of prevalence among adults [1]. This has led many researchers to hypothesize that humans can acquire immunity to *S. mansoni*, leading to partial resistance against reinfection [2]. Since the natural lifespan of *S. mansoni* worms is approximately 5–10 years [3],[4], the decline in prevalence coincides with the time at which worms acquired in early childhood would naturally begin to die in persons living in endemic areas. One theory holds that upon worm death, either naturally or as a result of treatment, critical schistosome antigens not normally or appropriately encountered by the host during chronic infection are released. The release of these antigens alters the immune response patterns that result from exposure to intact worms [5],[6], and it is hypothesized that these changes in immune responses are responsible for the increased resistance to reinfection [2].

We previously reported the age-independent development of immunological resistance to reinfection with *S. mansoni* in a cohort of adult males occupationally exposed, by washing cars in Lake Victoria, undergoing repeated cycles of reinfection and praziquantel-induced cure [7]. Resistance to reinfection by all three of the schistosome spcies that cause most human disease has been associated with both cellular [8],[9],[10] and humoral immune responses, most notably IgE in response to parasite-specific antigens [11]–[16]. In turn, variations in these immune responses have been related to factors such as age, stage of disease, and duration of infection [17]–[24].

More recently, we have expanded our studies to include a second cohort of men who are also exposed to infectious water through their occupation of harvesting sand in Lake Victoria. Upon discovering differences in the two cohorts in the number of treatments and cures needed before increased resistance to reinfection was demonstrated, we explored demographic and immunologic factors that may explain the discrepancies.

## Methods

### Study population

All participants in this study were adult males occupationally exposed to *S. mansoni* by washing cars or harvesting sand on the shores of Lake Victoria near Kisumu, Kenya. The car washers stand ankle- to knee-deep in the lake to wash cars that have been driven into the shallow water at the edge of the lake. Enrollment of car washers began in June 1995, and follow-up continued until January 2009. With the exception of the period between January 2000 and September 2003, enrollment of new car washers was continuous throughout the duration of the study, so follow-up time varies for each individual.

The sand harvesters stand waist- to chest-deep in the water to shovel sand off the bottom of the lake. After filling their boats with sand, they then transport the sand to shore and stand in the water at the edge of the lake while they unload the sand onto the shore. Recruitment of sand harvesters began in March 2005, and follow-up continued until January 2009. Both groups of men are ethnically homogeneous, with 90% of the car washers and 98% of the sand harvesters belonging to the Luo tribe.

**Figure 1:**
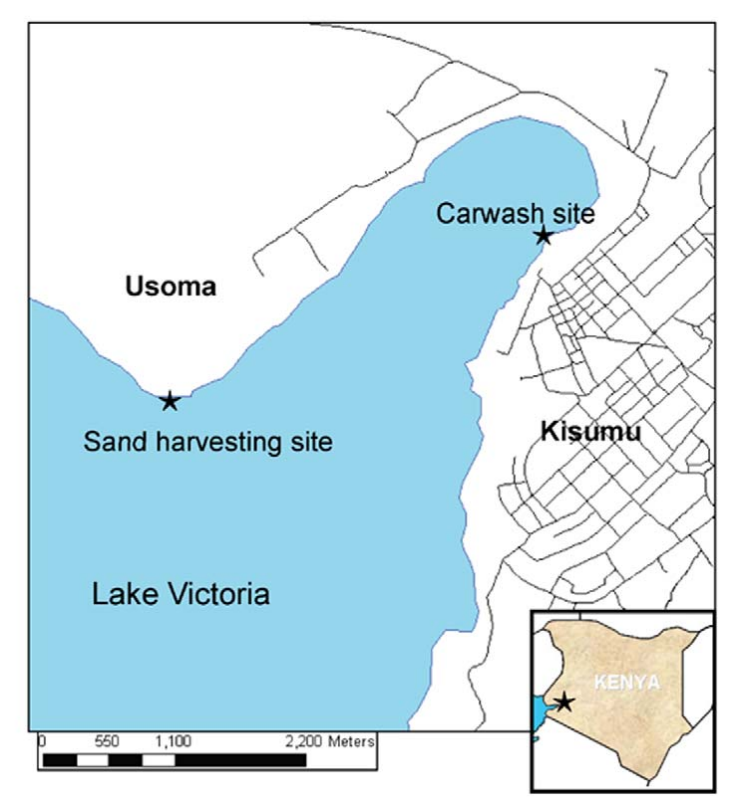
Map of area around Lake Victoria near Kisumu, Kenya, showing locations of carwash and sand harvesting sites.

The car washing and sand harvesting sites are shown in Figure 1. The carwash is adjacent to the city of Kisumu, and the site is a busy area also populated with fishermen, fish merchants, and various other vendors. Although located only 5.2 km around the lakeshore and 3 km across the lake, the sand harvesting site differs considerably as it is located off the shores of the small fishing village of Usoma, a rural community separated and distinct from the city of Kisumu. The presence of *S. mansoni*-infected *Biomphalaria sudanica* snails has been confirmed at both exposure sites [25],[26].

All study participants gave written informed consent prior to enrollment. Study procedures were approved by the institutional review boards of the University of Georgia and the Centers for Disease Control and Prevention, the Scientific Steering Committee of the Kenya Medical Research Institute (KEMRI), and the KEMRI/National Ethics Review Committee of Kenya.

### Patient follow-up

Upon enrollment, men were tested for *S. mansoni* eggs by the modified Kato-Katz method using two slides from each of three consecutive stool samples. Individuals positive for *S. mansoni* were treated with 40 mg/kg praziquantel (PZQ), and follow-up stool samples were taken 4–6 weeks later to assess for cure. If necessary, men were re-treated with PZQ until cure was demonstrated by three consecutive stool samples that were negative for schistosome eggs. Upon becoming stool negative, men were continually followed and retested for *S. mansoni* eggs at 4-week intervals. Each time a new infection was found, the study participant was treated with PZQ until he demonstrated cure.

Blood samples were taken every six months for subjects enrolled in 2003 or later and approximately yearly for car washers enrolled prior to 2003. Blood was tested for schistosome-specific antibodies, HIV-1 specific antibodies, and the ability of their peripheral blood mononuclear cells (PBMCs) to produce cytokines [7],[27]. The prevalence of malaria and soil-transmitted helminths in these populations was low. In the rare event malaria or soil-transmitted helminths were found the subjects were offered appropriate treatment.

Water exposure was measured by the number of cars washed or the number of days worked in the lake harvesting sand. Daily records of the number of cars washed by each car washer or the number of hours worked each day harvesting sand for each sand harvester were kept by on-site members of the carwash and sand harvester consortia who were employed as field workers for the present study. Since the number of hours spent in the water each day for sand harvesters was highly consistent (mean 5.3±0.9 hours), and sand harvesters likely receive most of their exposure to schistosomes as they are standing near the edge of the lake unloading the sand from their boats rather than when they are harvesting sand in waist- to chest-deep water away from the shore, we have chosen to use days worked rather than hours worked in water exposure calculations for the sand harvesters. Sand harvesters were given credit for one day of work for each day that they worked for at least one hour. It is important to note that one car washed is not equivalent to one day of work harvesting sand, thus direct comparisons between the two groups of men are not appropriate,

### Cytokine production and evaluation

Isolation of PBMCs and cell cultures were performed as previously described [28]. Briefly, PBMCs were separated from venous blood using the ficoll-hypaque technique. PBMCs were washed and resuspended in RPMI containing 5% AB+ normal human sera, antibiotics and L-glutamine. The cells were incubated with 10 µg/ml soluble worm antigen preparation (SWAP) or 5 µg/ml soluble egg antigens (SEA) for five days at 37C in 5% CO_2_ and the supernatant fluids collected. PBMC production of the cytokines interleukin (IL)-5, IL-10, IL-13, and IFN-γ in response to SWAP and SEA was measured by capture ELISA using commercially-available kits (R&D Systems, Minneapolis, MN) according to manufacturer's instructions. Cytokine production was only performed on blood samples obtained after October 2003.

### Antibody evaluation

Anti-SWAP IgE isotype ELISAs were performed on plasma from the venous blood samples as previously described [29],[30]. External positive and negative controls (EC) comprised of pooled samples of high responders and normal human serum (NHS) from non-endemic volunteers, respectively, were run on each plate. Anti-SWAP IgE values for each sample were standardized according to the following formula:
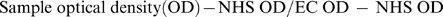



IgE-specific ELISAs against the recombinant antigens ‘tegument allergy like’ (TAL)-1 (formerly Sm22.6) and TAL-2 (formerly Sm21.7) [31] were performed on a subset of baseline samples from 23 car washers and 20 sand harvesters. TAL-1 and TAL-2 were cloned and purified as previously described [32],[33]. ELISA plates were coated with recombinant antigen at 2 µg/ml. Following incubation with plasma samples (1∶20 dilution), antigen-specific IgE binding was measured using directly conjugated mouse anti-human IgE (Southern Biotech, Birmingham, AL).

### Statistical methods

Since almost all measurements were non-normally distributed, the Wilcoxon rank sum test was used for group comparisons, and the Wilcoxon sign rank test was used for paired comparisons of the same subjects at different time points. An alpha level of 0.05 was considered statistically significant for all comparisons. All analyses were performed with GraphPad Prism 5 or SAS version 9.1.

The number of cars washed or days worked harvesting sand between each cure and reinfection was estimated in an accelerated failure time model with the LIFEREG procedure in SAS [34]. Each infection interval was defined as the time between the documentation of cure and subsequent reinfection. Thus, “interval 1” is the interval between the first cure after study entry and the first reinfection following the first cure, “interval 2” is the interval between the time of the second cure and second reinfection, and so forth. Interval number was entered into the model as a categorical variable with interval 1 as the reference category. Thus the length of each cure-to-reinfection interval was statistically compared to that of the first interval.

The LIFEREG procedure can accommodate failure time data that is right- or left-censored. The first interval was considered left-censored for subjects negative at study entry. Intervals during which the subject left the study or follow-up ended before reinfection occurred were considered right-censored. Censored observations accounted for 59 of 570 total intervals (10.4%) among the car washers and 30 of 144 total intervals (20.8%) among the sand harvesters. Intervals during which more than three months elapsed between the last negative stool and a subsequent positive stool were excluded from the analyses, though other intervals from that same subject could be included. Entire subjects were excluded from the analysis if they did not have at least one complete infection interval—i.e. left the study without ever becoming egg-negative or after the initial cure but before the first reinfection.

Because daily records of car washing activities are incomplete prior to 1999, subjects whose entire follow-up occurred before February 1999 are not included in this analysis. For subjects enrolled before February 1999 and followed further, the cure-to-reinfection intervals occurring after February 1999 are included, beginning with the numbered interval that the subject had reached at that point.

The final study population consisted of 120 car washers with a mean follow-up time of 74.4 months (range: 9.1–165.5) and 53 sand harvesters with a mean follow-up time of 37.9 months (range: 12.6–61.1). The mean number of cure-to-reinfection intervals was 6.5 (range: 1–18) and 3.0 (range: 1–8) for the car washers and sand harvesters, respectively.

For each car washer, the number of reinfections per 100 cars washed (RCW) during the at-risk time over the course of follow-up was calculated as an indication of relative resistance to *S. mansoni* reinfection. For the sand harvesters, this measure was calculated as the number of reinfections per 100 days worked harvesting sand (RDW) during the at-risk time over the course of follow-up. At-risk time is the time between cure and reinfection. Cars washed (or days worked) in the time between infection and cure are not included in the RCW or RDW calculations. As the RCW or RDW is averaged over the entire duration of follow-up, in theory those men who enter the study with a higher level of resistance or develop resistance over the course of the study will have a lower RCW or RDW than men who retain a high degree of susceptibility over the course of follow-up. For some analyses, car washers and sand harvesters are dichotomized based on the mean RCW or RDW of each respective group. For ease of discussion, men with a below-mean number of reinfections are referred to as “more resistant phenotype,” and men with an above-mean number of reinfections are referred to as “more susceptible phenotype.” Factors associated with having a more resistant phenotype were evaluated in a logistic regression model.

## Results

### Baseline characteristics

#### Demographics

Baseline characteristics of car washers and sand harvesters are given in Table 1. Sand harvesters were significantly older and reported working in the lake significantly more years prior to study entry than did car washers. Essentially all (98%) sand harvesters reported being born in Usoma, the lakeside village where they harvest sand. Conversely, the car washers are mostly from the city of Kisumu or emigrants from other areas of Kenya, and only 11% reported being born in a village near Lake Victoria. Initial mean egg counts were high (914 epg in the car washers and 876 epg in the sand harvesters) and were not significantly different between the two groups. The prevalence of HIV seropositivity was also high and similar in the two groups (18% versus 20%, Table 1).

**Table 1:** Demographic characteristics of car washers and sand harvesters at study entry.

	Car washers	Sand harvesters	p-value
Age in years [mean (std)]	24.5 (9.0)	27.7 (7.9)	0.0010
Years worked in lake [mean (std)]	5.7 (7.4)	11.1 (8.0)	<0.001
Born in lakeside village [n (%)]	9 (11.1)	51 (98.1)	<0.001
HIV positive [n (%)]	20 (17.9)	11 (21.2)	0.6169
Eggs per gram feces [mean (std)]	914 (1013)	876 (1064)	0.9764

#### Cytokine production

**Figure 2:**
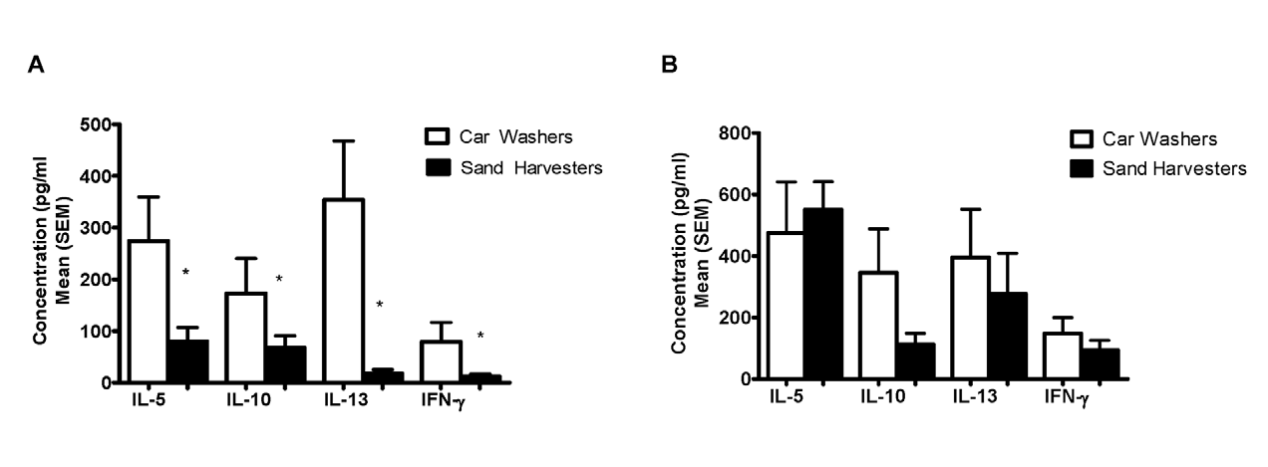
Baseline cytokine responses amongst the car washers and sand harvesters. Responses to SEA (A) and SWAP (B). *p<0.05 for difference between mean cytokine concentration produced by PBMCs from car washers and sand harvesters.

**Figure 3:**
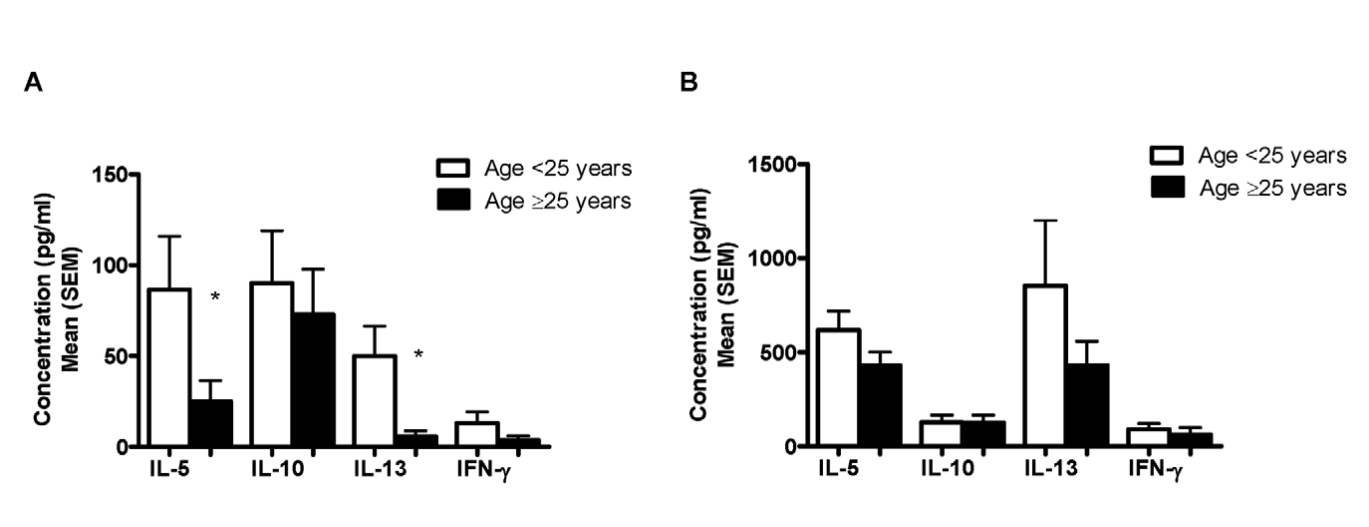
Baseline cytokine responses by age amongst the sand harvesters. Responses to SEA (A) and SWAP (B). *p<0.05 for difference between mean cytokine concentration in men aged <25 years versus men ≥25 years.

Figure 2 shows baseline pre-treatment cytokine production in response to SEA (Figure 2a) and SWAP (Figure 2b) by the PBMCs from car washers and sand harvesters. SEA-stimulated production of all four cytokines was significantly higher in car washers than in sand harvesters. No significant differences between the two groups were seen in cytokine production in response to SWAP. Among the sand harvesters, men under age 25 showed higher levels of SEA-stimulated IL-5 and IL-13 than men aged 25 years and older (Figure 3a). No differences by age were seen in SWAP-stimulated cytokine production (Figure 3b). While we did not have baseline cytokine data on any car washers over age 25, when <25 year old car washers were compared with <25 year old sand harvesters, cells from young car washers responded significantly higher to SEA, but not SWAP, by IL-5 and IL-13 production than did young sand harvesters (p = 0.0028 and 0.0087, respectively).

#### IgE antibody responses

**Figure 4:**
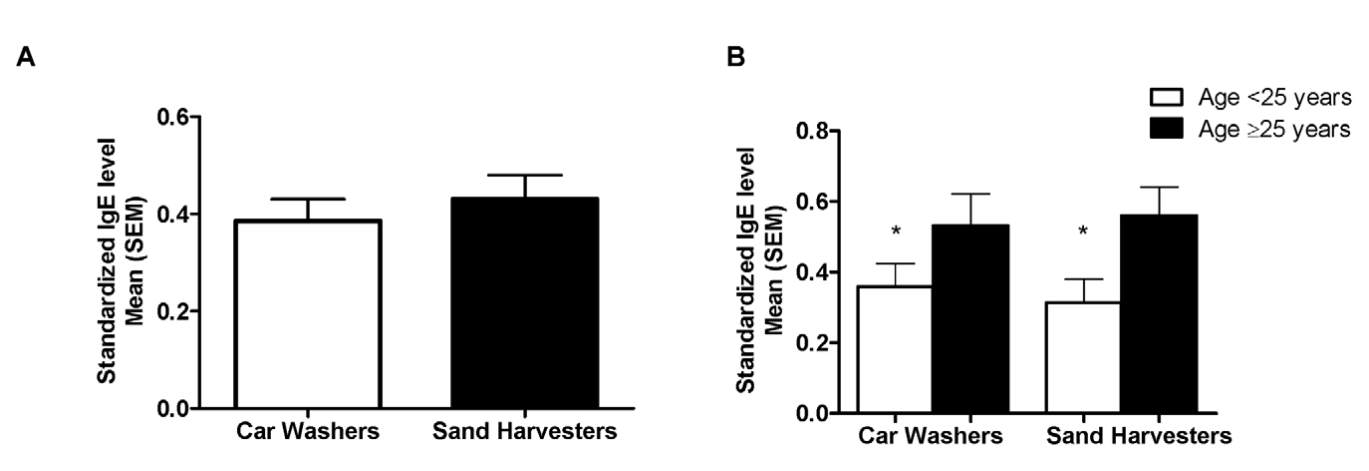
Baseline anti-SWAP IgE responses. Responses in car washers versus sand harvesters (A) and by age within each cohort (B). *p<0.05 for difference between mean cytokine concentration in men aged <25 years versus men ≥25 years.

**Figure 5:**
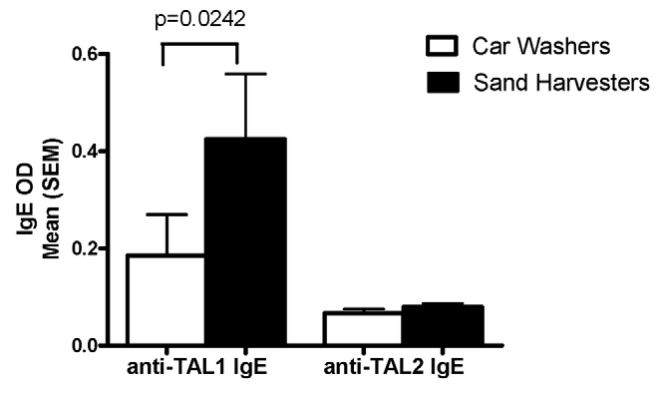
Baseline anti-TAL-1 and anti-TAL-2 IgE responses in car washers and sand harvesters.

Serum IgE levels to a crude worm antigen preparation (SWAP) and two recombinant antigens (TAL-1 and TAL-2) were measured in both cohorts. Overall, pre-treatment anti-SWAP IgE levels did not differ between the two groups (Figure 4a). However, in both car washers and sand harvesters, men aged 25 years and older expressed higher pre-treatment levels of anti-SWAP IgE than did younger men (Figure 4b). Pre-treatment anti-TAL-1 and anti-TAL-2 IgE levels in car washers and sand harvesters are shown in Figure 5. At baseline, sand harvesters expressed significantly higher mean levels of anti-TAL-1 IgE than did car washers, while mean anti-TAL-2 IgE responses were not significantly different and were low in both groups.

### Development of resistance

**Figure 6:**
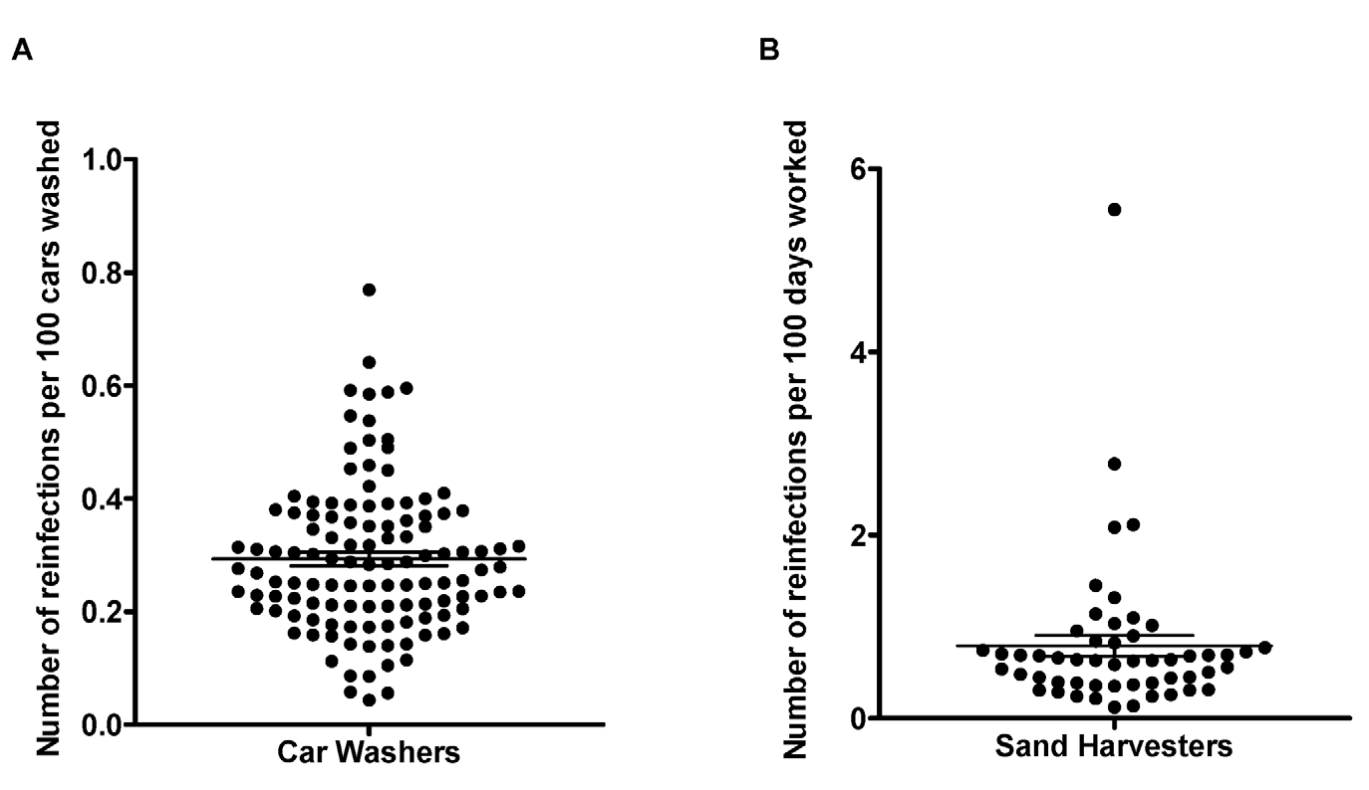
Indices of resistance in car washers and sand harvesters. Distribution of the number of reinfections per 100 cars washed (RCW) in car washers (A) and number of reinfections per days worked (RDW) in sand harvesters (B). Horizontal lines represent mean (± standard error of the mean).

The RCW or RDW for each car washer and sand harvester is plotted in Figure 6. The mean RCW for the car washers was 0.29 infections per 100 cars washed, with the individual RCWs uniformly distributed around the mean. Conversely, the sand harvesters exhibited a skewed pattern of resistance indexes, with the majority of the individual RDWs concentrated below the mean of 0.79 infections per 100 days worked, and only a few men with higher outlying RDWs.

**Figure 7:**
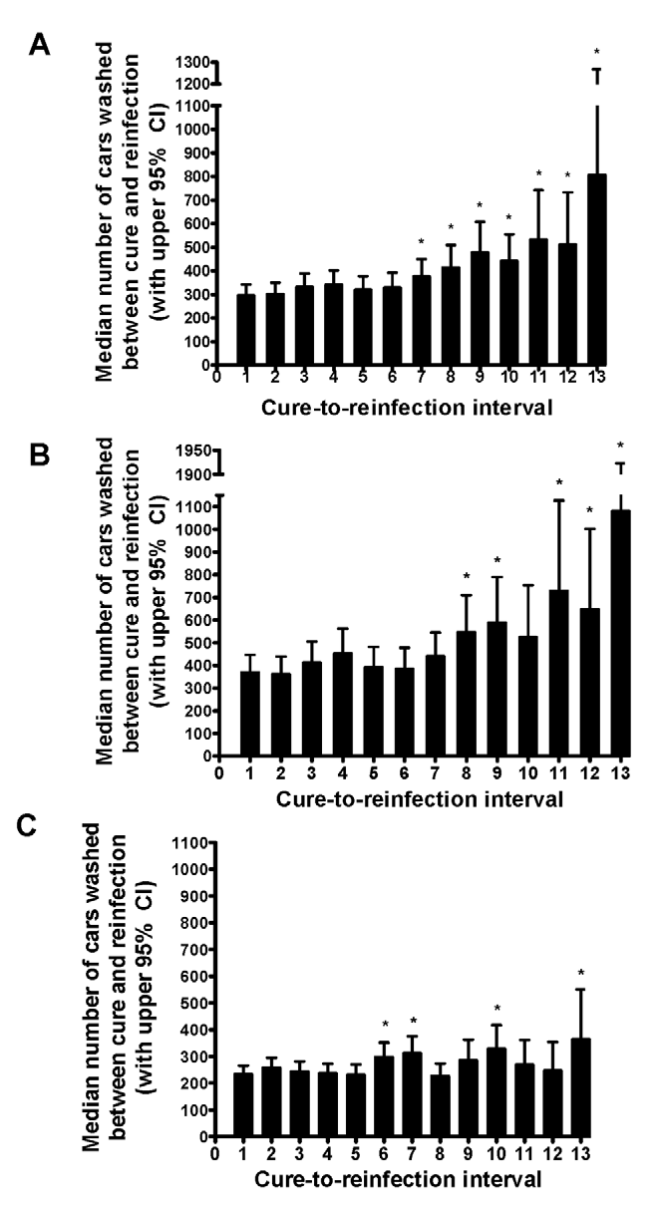
Median number of cars washed between each cure and subsequent reinfection in car washers. The median number of cars washed between each cure and subsequent reinfection (and corresponding 95% confidence intervals) was estimated with the LIFRREG procedure in SAS. Figures represent total cohort of car washers (A) and cohort stratified into resistant (B) and susceptible (C) phenotypes. “Cure-to-reinfection interval 1” is the interval between the first cure after study entry and the first reinfection following the first cure, “cure-to-reinfection interval 2” is the interval between the second cure and second reinfection, and so forth. *Length of interval significantly greater than first cure-to-reinfection interval.

Figure 7 shows the median number of cars washed in the intervals between each successive cure and reinfection. The figure depicts all car washers together (Figure 7a), and also stratified into more resistant (Figure 7b) and more susceptible (Figure 7c) phenotypes based on being below or above the mean RCW, respectively. For the entire cohort of car washers, the number of cars washed before reinfection was relatively stable until the seventh cure, at which point the number of cars washed between cure and reinfection begins to progressively increase with each successive cure. In the seventh cure-to-reinfection interval, and each interval thereafter, the number of cars is significantly greater than the number of cars washed in the interval between the initial cure and the first reinfection. When the car washers were stratified based on the RCW, those with the more resistant phenotype (Figure 7b) showed a pattern of increasing cure-to-reinfection intervals similar to that seen in the overall cohort. With the exception of interval ten (p = 0.0922), the median number of cars washed in each cure-to-reinfection interval after the eighth cure in the more resistant phenotype group was significantly greater than the initial interval (p-value range: 0.0005–0.0195). However, a pattern of increasing number of cars per cure-to-reinfection interval was not seen in the group of men with the more susceptible phenotype (Figure 7c). While some later intervals were significantly greater than the initial interval, overall these men did not, by the end of the study, exhibit a consistent pattern of increased resistance to reinfection upon repeated cures.

**Figure 8:**
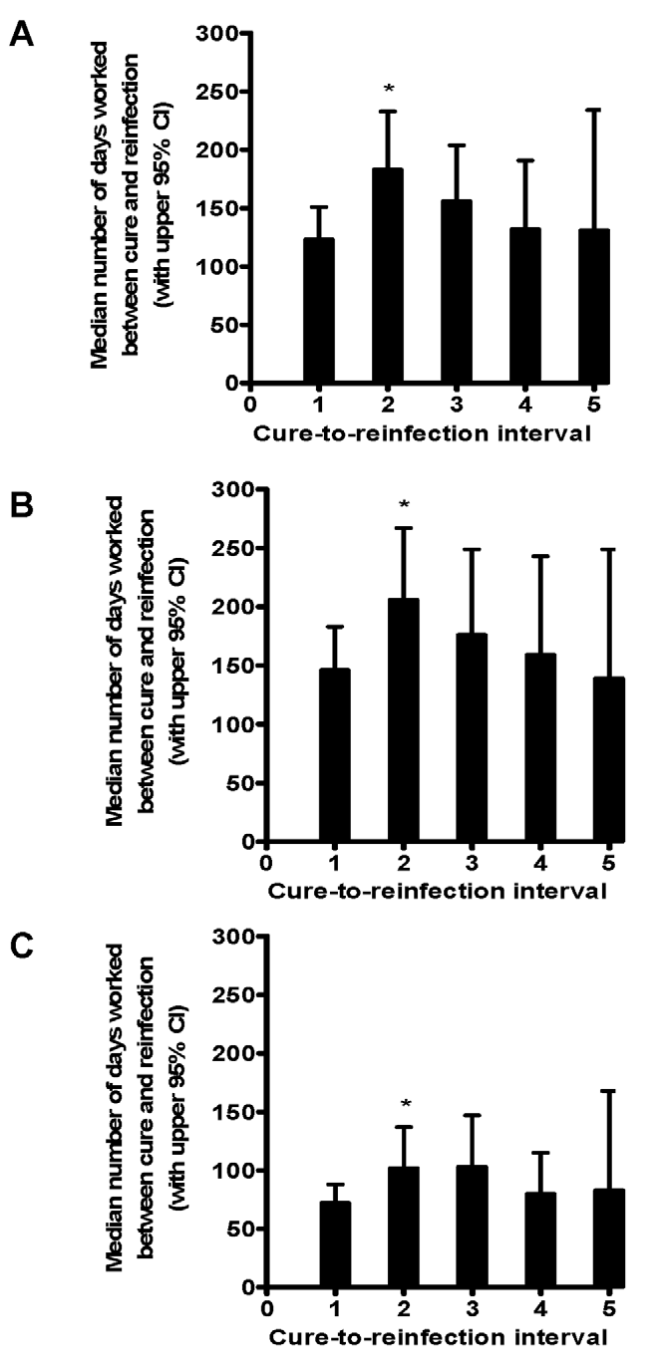
Median number of days worked between each cure and subsequent reinfection in sand harvesters. The median number of days worked between each cure and subsequent reinfection (and corresponding 95% confidence intervals) was estimated with the LIFRREG procedure in SAS. Figures represent total cohort of sand harvesters (8a) and cohort stratified into resistant (8b) and susceptible (8c) phenotypes. “Cure-to-reinfection interval 1” is the interval between the first cure after study entry and the first reinfection following the first cure, “cure-to-reinfection interval 2” is the interval between the second cure and second reinfection, and so forth. *Length of interval significantly greater than first cure-to-reinfection interval.

The median number of days worked in Lake Victoria between each cure and reinfection for all sand harvesters are shown in Figure 8a. The number of days in the interval between the second cure and second reinfection was significantly increased relative to the initial interval (p = 0.0118). Thus, as opposed to the car washers, the increase in resistance occurred in the sand harvesters after having experienced only two previous cures. This pattern was true for men with both more resistant and more susceptible phenotypes (Figures 8b–8c), though the more susceptible men started with a lower initial days worked to reinfection, and days to reinfection remained lower throughout follow-up. Although the graph appears to show a trend towards increased susceptibility after three previous cures, intervals 3–5 do not have significantly fewer days worked than interval two, and the apparent decrease is likely due to low numbers of subjects and high numbers of censored observations in intervals three and above.

#### Factors associated with a more resistant phenotype

Associations between baseline factors and having a more resistant phenotype based on the RCW or RDW are shown in Table 2. In univariate analysis, car washers who were HIV positive at study entry had 0.4 times reduced odds of having a more resistant phenotype than HIV negative men. Neither their age at baseline nor the number of years they had worked in the lake was associated with being in the resistant or susceptible group. The effect of HIV remained in the multivariate analysis, though the estimate became more unstable due to the addition of extra degrees of freedom to the model. Amongst the sand harvesters, age ≥25 years and working in the lake at least 10 years prior to study entry were associated with having a more resistant phenotype in univariate analysis, while HIV status was not associated with resistance. However, the effect of prior time worked in the lake was no longer present in multivariate analysis, and age is the only independent predictor of resistance in this cohort. For sand harvesters aged 25 years and older, the odds of having greater resistance to reinfection were increased by 4.5 times compared to younger men.

**Table 2:** Odds ratios (95% confidence intervals) for associations between baseline characteristics and having a more resistant phenotype.

Variable	Car washers		Sand harvesters	
	Univariate	Multivariate^a^	Univariate	Multivariate^a^
Worked in lake >10 yrs	1.5 (0.5, 4.9)	2.6 (0.4, 16.1)	**3.7 (1.0, 13.9)**	1.7 (0.3, 9.6)
Age >25 yrs	1.1 (0.4, 2.9)	0.6 (0.1, 2.7)	**4.5 (1.2, 16.9)**	4.3 (0.8, 24.5)
HIV positive	**0.4 (0.1, 1.0)**	0.4 (0.1, 1.3)	1.0 (0.2, 4.4)	1.4 (0.3, 7.2)

More resistant phenotype is defined as experiencing a below-average number of reinfections (<0.29 reinfections/100 cars washed or <0.70 reinfections/100 days worked harvesting sand).

a Adjusted for the other variables listed in the table.

#### Changes in anti-SWAP IgE levels over time

**Figure 9:**
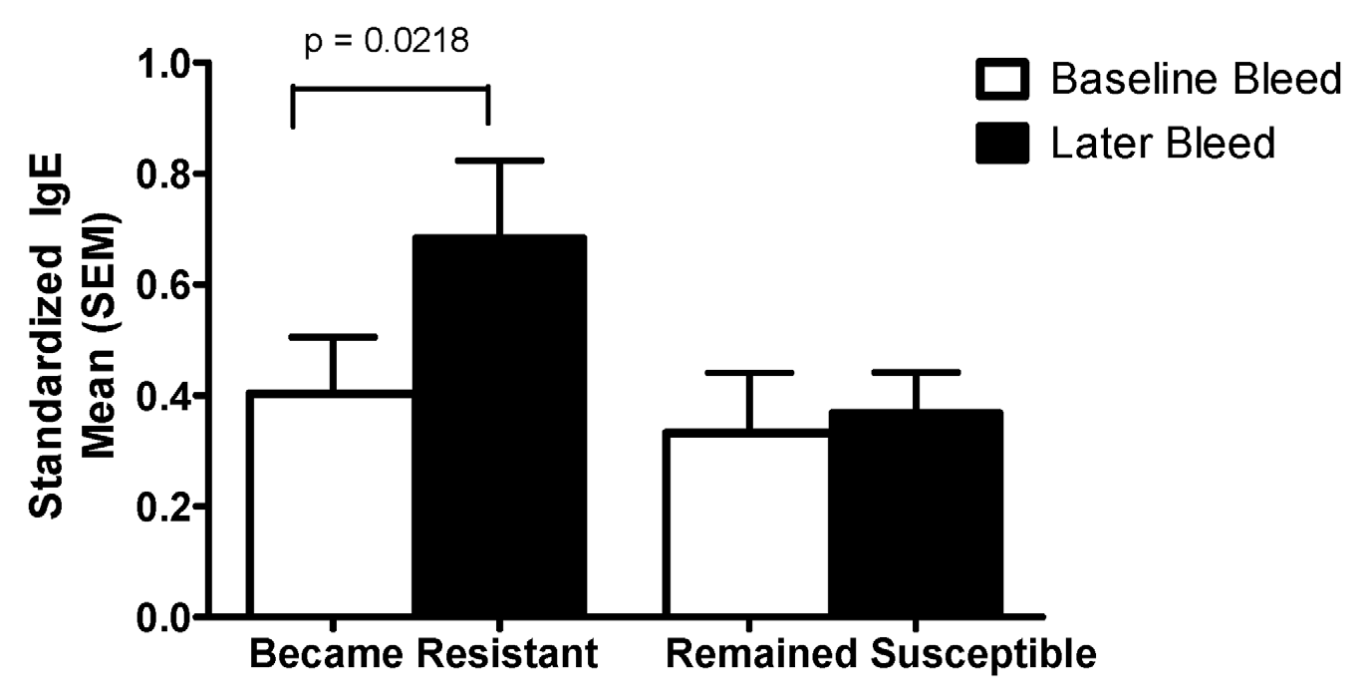
Change in anti-SWAP IgE accompanying the development of resistance to *S. mansoni* reinfection. This figure depicts the change in anti-SWAP IgE over time among car washers that developed resistance to *S. mansoni* reinfection and those that remained susceptible to reinfection. In the group that developed resistance, the later bleed is the first bleed after the cure for which the length of cure-to-reinfection intervals surpassed 450 cars. In the group that remained susceptible, the later bleed is the final bleed collected for the study, after subjects had received a mean of 12±6.7 PZQ treatments.

In Figure 9, anti-SWAP IgE levels are shown at two timepoints for the group of car washers that began the study having washed <450 cars between the first cure and reinfection but consistently washed more than 450 cars before becoming reinfected over the course of follow-up (labeled “became resistant”), and the group of car washers that became reinfected after washing <450 cars at the beginning of the study and never required washing >450 cars to become reinfected over the duration of the study (labeled “remained susceptible”). In both groups, the baseline bleed was taken at study entry before any treatment was administered. In the group that developed resistance, the later bleed depicted in the figure is the first bleed after the cure for which the length of cure-to-reinfection intervals surpassed 450 cars. In the group that remained susceptible, the later bleed is the final bleed collected for the study. In the men who became more resistant to reinfection after experiencing multiple reinfections and cures, the level of anti-SWAP IgE significantly increased between baseline and the time at which development of resistance was first evidenced. No significant changes in anti-SWAP IgE over the course of follow-up were observed in men who did not show evidence of development of resistance to *S. mansoni* by the end of the study. While the men who remained susceptible were more likely to be *S. mansoni* positive at the time of the later bleed than those men who developed resistance (71% vs 13%, p<0.001), infection status at the time of the later bleed did not affect the magnitude of change in anti-SWAp IgE between baseline and later bleeds.

No significant increases in anti-SWAP IgE over time were observed in either group of sand harvesters.

## Discussion

Previous research by our group has shown that among men similarly exposed to *S. mansoni* by virtue of their occupation as car washers in infectious waters of Lake Victoria, a portion of the men developed resistance to reinfection after multiple rounds of cures and reinfections, while others remained susceptible despite equal or greater numbers of cures and reinfections [7]. We now show that the same observation holds true with a modified definition of resistance, based on exposure rather than time-to-reinfection, and after the addition of another cohort of men at the same carwash and additional follow-up of the original cohort. If car washers were maximally immune or non-immune at study entry, we would not have observed a progressively increasing number of cars before each reinfection as we did in many of the cohort, suggesting that these men are actively developing resistance. Those car washers who developed resistance began to do so after experiencing an average of seven previous cures.

However, a different pattern of the development of resistance emerged in a different cohort of men who receive daily exposure to schistosomes by harvesting sand in the lake just three km across the lake from the car washing site. In these men, the interval between cure and reinfection significantly increased after only two previous cures, after which point there were no further increases in the number of days worked between cure and reinfection, suggesting that no further increases in resistance occurred.

The numerical values of reinfections per 100 cars washed and reinfections per 100 days worked harvesting sand are not directly comparable as the *S. mansoni* transmission situation is different for each cohort. The sand harvesters spend on average 5.3 hours in the lake each work day, while the car washers wash an average of 3.2 cars per work day. However, the water at the car washer site is probably more heavily contaminated with *S. mansoni* cercariae, as the prevalence of infection in *B. sudanica* snails collected at the car wash site is higher than prevalence in snails collected at the sand harvesting site in Usoma [26]. Also, much of the sand harvesters' time is spent in deeper water, away from the shore where snails are not as likely to be present, so the majority of their cercarial exposure likely occurs during the time they are unloading sand onto the shore. Despite the differences in exposure between cohorts, the definition of resistance for each cohort is valid for comparisons within that cohort, and overall patterns of resistance should be comparable between the two cohorts.

The car washers exhibited a wide range of overall resistance levels according to the distribution of number of reinfections per 100 cars washed, which were fairly symmetrically distributed from the low to the high end of the spectrum. Although some car washers in the more susceptible group appeared to develop resistance after multiple cures and some might have eventually become resistant with longer follow-up, the general pattern observed was a gradual increase in resistance among those men who experienced a below-mean number of reinfections and no apparent consistent increase in resistance among men who experienced an above-mean number of reinfections. In contrast, the distribution of number of reinfections per 100 days worked for the sand harvesters was much less uniform, with the majority of the sand harvesters clustering towards fewer reinfections, indicating that a majority of the cohort entered the study with similar relatively high levels of resistance. Albeit to a much less degree than those in the more resistant group, even those relatively more susceptible sand harvesters exhibited an increase in time-to-reinfection after two previous cures.

The different histories of *S. mansoni* exposure in these two groups of men prior to study enrollment likely explain the differences in development of resistance upon multiple rounds of treatment and reinfection. The car washers reported working in the lake a mean of 5.7 years, while the sand harvesters had worked in the lake for a mean of 11.1 years. Moreover, while the majority of car washers were lifelong residents of the city of Kisumu or immigrants from other areas of Kenya, almost all of the sand harvesters were born in Usoma, the lakeside village where they now harvest sand. *S. mansoni* infection has been seen in children in Usoma as early as one year of age, with >90% becoming positive for antibodies to schistosomes by age 10 (J. Verani, unpublished data). A similar situation has been reported among children in fishing villages along the Ugandan shoreline of Lake Victoria, where Odogwu and colleagues found *S. mansoni* infection in 25% and 86% of children aged <3 years in two endemic villages [35]. Thus, men from Usoma likely had exposure to the lake as children long before they began working as sand harvesters, were probably initially infected with *S. mansoni* at an early age, and had likely experienced the natural death of worms multiple times prior to being treated as part of this study. In contrast, *S. mansoni* infections present at study entry in car washers likely represent more recent infections, and they had likely experienced the death of no or few worms prior to treatment with praziquantel.

These two groups of occupationally exposed adult males also differed considerably in their immune response patterns to schistosome antigens, and these differences are also likely explained by their different histories of exposure to *S. mansoni*. The baseline immune responses are suggestive of more recent infections in car washers. PBMC cytokine production in response to SEA at the time of enrollment was higher in car washers than amongst sand harvesters by all four measured cytokines. High responses to SEA have been associated with early *S. mansoni* infection, and these responses then decrease as infection becomes more chronic and exposure to constantly released egg antigens leads to development of immunoregulatory mechanisms [19],[20],[22],[23].

Similar to other researchers who have shown no difference in humoral responses to crude worm antigens in patients with early and chronic schistosomiasis [19], baseline anti-SWAP IgE responses did not differ between our cohorts. However, in both car washers and sand harvesters, older men had significantly higher levels of anti-SWAP IgE than did younger men, independent of exposure history. While increases in parasite-specific IgE with increased age are usually attributed to longer exposure to infection, Naus et al also reported increased IgE responses against schistosome worm antigens in older age groups in an immunologically naïve immigrant population recently arrived to an *S. mansoni*-endemic area of Kenya, suggesting that the increase may be innately age-related and not dependent on duration of schistosome infection [18].

Although baseline differences between car washers and sand harvesters were not seen in IgE responses to the heterogeneous worm antigens present in SWAP, differential IgE responses to the recombinant *S. mansoni* antigens TAL-1 and TAL-2 were observed between the two groups. Fitzsimmons et al have shown that TAL-1 expression is concentrated primarily in the adult worm, while TAL-2 is expressed on all life cycle stages, including miracidia, cercariae, and eggs [33]. Levels of anti-TAL-1 IgE antibodies were increased after treatment of *S. mansoni* infected individuals in the Fitzsimmons et al study, while anti-TAL-2 IgE antibodies were unchanged by treatment. The authors hypothesized that TAL-1 worm antigens are sequestered during active infection and are only released upon worm death. Conversely, the immune system is continuously exposed to TAL-2 due to the constant release of eggs during *S. mansoni* infection [32], thus leading to down regulation of responses to TAL-2. The current finding of higher pretreatment levels of anti-TAL-1 IgE in sand harvesters than in car washers and similarly low anti-TAL-2 responses in both groups fits this hypothesis. As the natural lifespan of an adult *S. mansoni* worm is approximately 5–10 years [3],[4], the car washers had likely not been exposed to any or many dying worms before receiving PZQ treatment as part of the current study, while the sand harvesters had likely already experienced multiple episodes of naturally dying worms, based on exposure since early childhood.

Car washers who were HIV positive at study entry were less likely to develop resistance over the course of follow-up than were men who were HIV negative. We previously reported that patients with schistosomiasis and HIV coinfection had significantly lower production of the cytokines IL-4 and IL-10 than schistosome-infected persons who were HIV negative [28]. Other researchers have reported an association between IL-4 production in response to schistosome antigens and increased resistance to reinfection with *S. mansoni* [8], *Schistosoma haematobium* [9], and *Schistosoma japonicum* [10]. HIV infection was not related to the ability to develop resistance in the sand harvesters, most probably because they had already been infected with and developed protective immune mechanisms against schistosomes prior to becoming infected with HIV as adults. While neither age nor number of years worked in Lake Victoria prior to study entry were associated with resistance among the car washers, only age was independently predictive of a resistant phenotype among the sand harvesters. As most sand harvesters likely had lake exposure since childhood before they began working harvesting sand, length of time worked in the lake became insignificant in the analysis after adjustment for age, as age is a better predictor for duration of water exposure in this group.

Many previous studies have shown various immune responses to be correlated with resistance to reinfection with all three species of schistosomes, most commonly the production of parasite-specific IgE [11], [14]–[16]. While we did not find any baseline antibody or cytokine responses to be predictive of the ability to develop resistance among the car washers, this was not unexpected given that a change in resistance did not become apparent until the men had experienced on average seven previous cures. However, among those car washers that did eventually demonstrate an increase in resistance against reinfection, we have documented increases in anti-SWAP IgE production that parallel the development of resistance. Increases in anti-SWAP IgE production did not occur in those who remained susceptible. We did not see a similar increase in anti-SWAP IgE as the interval between cure and reinfection increased in sand harvesters.

In conclusion, we have again demonstrated that resistance to reinfection with *S. mansoni* can be acquired or augmented by adults after multiple rounds of reinfection and PZQ-induced cure. However, we now also show that the ability to acquire this resistance and the rate at which resistance is acquired is markedly different in two populations within close geographic proximity to one another that share high levels of occupational exposure to *S. mansoni* infested water. These differences are likely attributable to differences in history of exposure to *S. mansoni* infection and their resulting immunologic status at baseline. As many conflicting results have been reported in the literature regarding immunologic parameters associated with the development of resistance to schistosome infection, these factors should be considered in the design of future immuno-epidemiologic studies and eventual vaccine study design.
